# Physical Rehabilitation Post-surgery in a Distal Femur Fracture Post Removal of Implant

**DOI:** 10.7759/cureus.51358

**Published:** 2023-12-30

**Authors:** Hrutuja J Karekar, Aditi Akhuj, Swapnil U Ramteke

**Affiliations:** 1 Sports Physiotherapy, Ravi Nair Physiotherapy College, Datta Meghe Institute of Higher Education and Research, Wardha, IND

**Keywords:** treatment, surgical intervention, range of motion, external fixator, comminuted distal femur fracture, physical therapy

## Abstract

A complicated orthopaedic injury known as a comminuted distal femur fracture occurs when the lower portion of the femur bone breaks into several pieces close to the knee joint. Distal femur fractures are more commonly caused by injuries to the supracondylar and intercondylar regions. Managing comminuted distal femur fractures is a significant clinical challenge. The age of the patient, the condition of the bone, any concomitant injuries, and the level of comminution all influence the treatment plan. Handling distal femur fractures that have comminuted presents a major clinical challenge. One of the possible treatment options may be surgical intervention using techniques such as open reduction and internal fixation (ORIF), total knee arthroplasty (TKA), or external fixation. In this article, we describe a 32-year-old man whose primary complaints were pain, the inability to bear weight on the afflicted side, and a comminuted distal femur fracture. He was operated with ORIF. After the removal of the implant, restoring a typical range of motion (ROM) and relieving discomfort were the main objectives of physical therapy. Physical rehabilitation following implant removal aims to optimize functional outcomes, restore joint mobility, and enhance muscular strength. This process involves a multidisciplinary approach, integrating the expertise of orthopaedic surgeons, physiotherapists, and rehabilitation specialists. Emphasis was placed on early mobilization, proprioceptive training, and tailored exercise regimens to address specific deficits related to the previous implant presence. The patient's recovery was aided by the physiotherapy routines.

## Introduction

A distal femur fracture pertains to a fracture occurring in the distal region of the femur. These fractures are relatively uncommon but can be severe and challenging to manage. One of the most widely used fracture classification schemes is the Arbeitsgemeinschaft Osteosynthesefragen (AO) classification of distal femoral fractures [1,2]. This is a severe injury, and realignment and stabilization of the bone frequently need surgical intervention [3]. Physical therapy may be necessary during the long recovery process from these fractures in order to regain function in the lower part of the leg and knee [4,5]. Fractures of the femur's distal portion can differ in location and severity. They may be categorized as supracondylar fractures that occur just above the knee joint. Intercondylar fractures involve the bony prominences within the knee joint. Condylar fractures affect the rounded parts of the femur at the knee [6]. Adults with bimodal distribution experience distal femur fractures [7]. Usually, younger male patients come in after vigorous events like car crashes. Senior people typically come up after low-energy incidents, such as falls from great heights. These patients frequently have significant comorbidities that impact their ability to function, heal, and survive as elderly patients [8]. The issue in the paediatric population can be the long-term effects of early joint injury and poorly managed intra-articular fractures. Treatment for these complex fractures has a poor prognosis as there is an increase in age [9].

The portion at the lower end of the femur, spanning about 15 cm, is commonly denoted as the distal femur and is located between the knee's articular surface and the metaphyseal-diaphyseal junction [10]. Injuries to the knee caused by flexion are often directly attributed to dashboard impacts in automobile accidents [11]. The location of a distal femur fracture in relation to the adductor tubercle determines the specific deforming forces at play. Typically, the adductor magnus, once shortened by the hamstrings and extensor mechanism, tends to shape the fracture into a varus. Simultaneously, the two heads of the gastrocnemius muscle extend the distal fragment, particularly when the fracture apex is angled posteriorly [12]. The rotation and division of the two heads can lead to patterns of intercondylar split fractures in the distal condylar fragmentation. Individuals with a history of multiple traumas might also face concurrent tibial fractures. In cases where distal femur fractures are caused by gunshot wounds, high-energy trauma, or open injuries, the risk of vascular damage becomes a significant concern [13].

Fractures occurring in the distal femur account for approximately 3-6% of all femoral fractures and constitute less than 1% of all fractures [14]. In a study, it was found that 80% of patients aged 35 years or older who experienced a distal femur fracture due to mild trauma exhibited generalized osteopenia. Reports indicate that distal femur fractures may occur with a risk ranging from 0.3% to 5.5% after primary total knee arthroplasty (TKA) and up to 30% after revision knee arthroplasty [15]. The objective of physical therapy following a distal femur fracture is to restore the patient to baseline function and prevent complications. To prevent post-surgery stiffness, patients should gradually increase their knee's range of motion (ROM). The duration of non-weight bearing, toe touch weight bearing, or partial weight bearing on the injured extremity may vary based on intraoperative stability, ranging from four to eight weeks. It is crucial to raise awareness of the condition to ensure effective treatment and rehabilitation. Intensive post-surgery physical therapy includes cardiovascular exercises, mobility/gait activities, and a series of progressive range-of-motion exercises aimed at stabilizing and healing the fracture.

## Case presentation

Patient information 

A patient with the age of 32 years met with a road traffic accident and was brought to hospital. He was examined by the orthopaedic surgeon and was recommended surgery for the left side after obtaining an X-ray that revealed a comminuted displaced distal end femur fracture. In terms of pain and ROM, the patient seemed to be doing well following his open reduction and external fixation procedure. On September 2023, following two months of surgery, the patient visited Acharya Vinobha Bhave Rural Hospital (AVBRH), a hospital care facility, for the implant's removal. He complained of left knee pain. An X-ray scan was performed following a consultation with the orthopaedic surgeon, which identified the infection in the implanted knee.

Clinical findings

Before conducting the physical examination, the patient provided both written and verbal consent. Upon observation, the patient was observed lying in a supine position. While the normal ROM was noted in the right limb, active movements in the left limb were not possible. The bending of the left knee was painful and restricted. Flexion deformity was seen, but no swelling was observed in the left knee. The knee's ROM was limited due to stiffness present in the left knee (Table 1). The patient also complained of difficulty in walking. On a pain rating scale of 1 to 10, the pain was noted to be 9. Pre-rehabilitation lower limb strength is shown in Table 2. We noticed tension in soft tissue in the left lower limb's gluteus and thigh muscles.

**Table 1:** ROM (in degrees) ROM: Range of motion

Joint movement	Pre-rehabilitation	
	Left	Right
Knee flexion	0°-15°	0°-120°
Knee extension	0°-10°	0°-120°
Ankle plantar flexion	0°-30°	0°-45°
Ankle dorsiflexion	0°-15°	0°-25°

**Table 2:** MMT MMT: Manual muscle testing; NA: Not Assessable

Muscles	Pre-rehabilitation	
	Left	Right
Hip flexors	NA	4/5
Hip extensors	NA	4/5
Hip abductors	NA	4/5
Hip adductors	NA	4/5
Knee flexors	NA	4/5
Knee extensors	NA	4/5
Ankle dorsiflexors	NA	4/5
Ankle plantar flexors	NA	4/5

Radiological findings 

**Figure 1:**
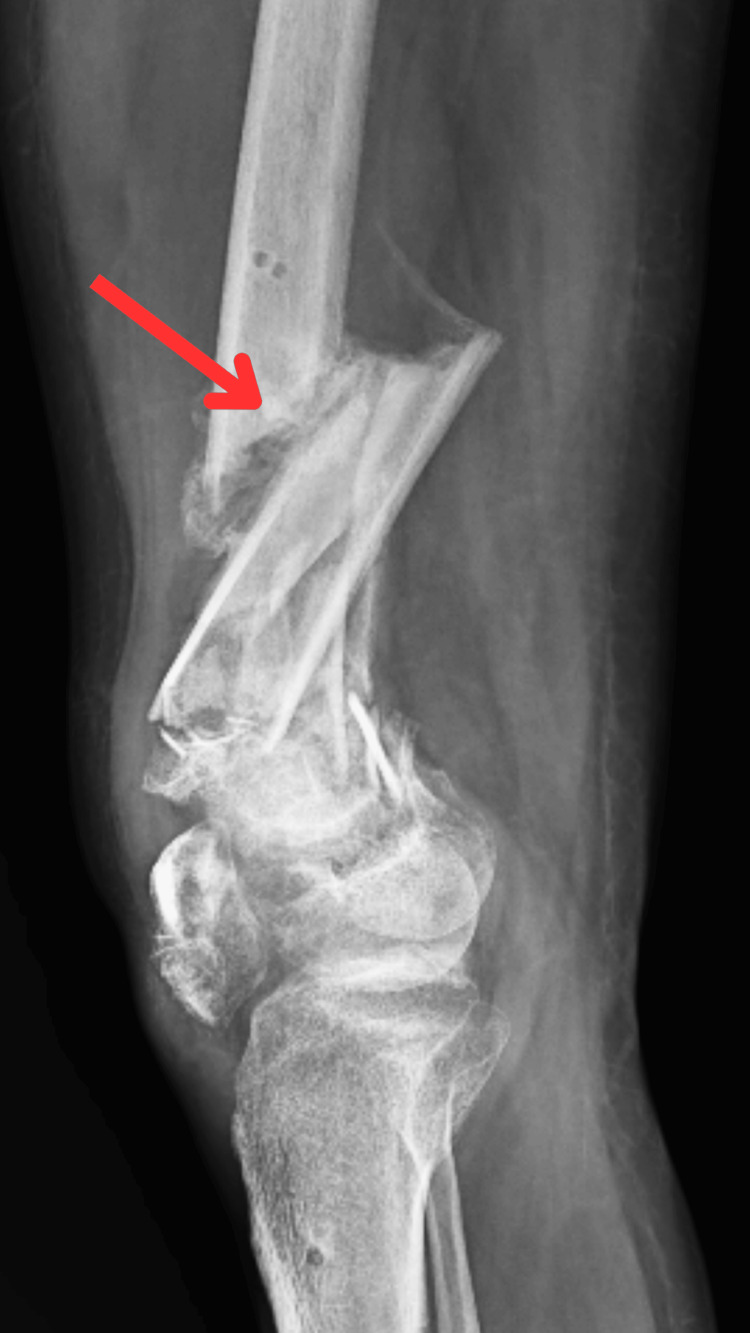
X-ray of comminuted distal femur fracture (lateral view) The red arrow points to the fracture site.

**Figure 2:**
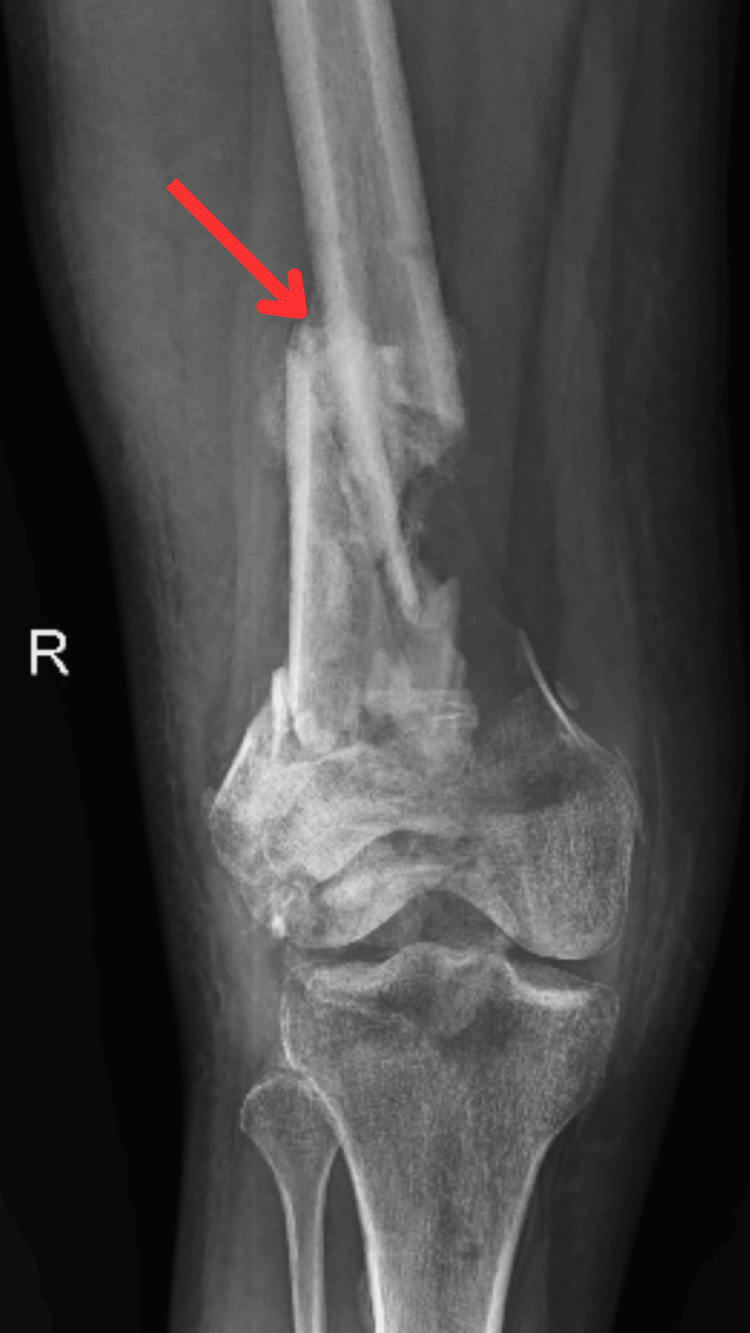
X-ray of comminuted distal femur fracture (anteroposterior view) The red arrow points to the fracture site.

Radiography scans were done to assess the condition. A pre-operative X-ray is displayed in Figures 1, 2 which shows an X-ray of comminuted distal femur fracture in lateral view and anteroposterior view, respectively, with the fracture site indicated by the arrow.

Physiotherapy intervention

Treatment goals were to educate the patient about the condition and to acquire the cooperation and consent of the patient and his family members, to avoid deformity and complications after surgery, to reduce pain at the site of the fracture, to aid in the flexibility of the hip and knee joints, to regain the strength of affected muscles, to start early weight bearing on the affected limb, to improve the activities of daily living (ADLs).

Week 1

In the initial week of treatment, the patient received education regarding their medical condition and the potential benefits of physical therapy. We began with achieving a full ROM in the ankle while introducing mild flexion in the left knee and hip. Isometric exercises were prescribed to target the quadriceps and gluteal muscles. The rehabilitation process commenced with non-weight bearing, assisted by a walker and utilizing a three-point gait.

Week 2 to 4

In the second week, we initiated active and active-assisted movements for the hip, knee, and ankle. Additionally, isotonic and isometric exercises for the hip, knee, and ankle were introduced. Non-weight bearing continued, supported by a walker and utilizing a three-point gait.

Week 4 to 8

By the fourth week of the treatment, active heel slides and 90 degrees of hip flexion were performed by the patient. Bedside sitting with their leg hanging down with the unaffected extremity supporting the affected extremity. Assisted and self-resistive exercise for the hip and knee was started. Four-point kneeling from a prone lying position was initiated and then progressed to knee walking. With the help of a walker and three-point gait, partial weight bearing is initiated.

After Week 8

**Figure 3:**
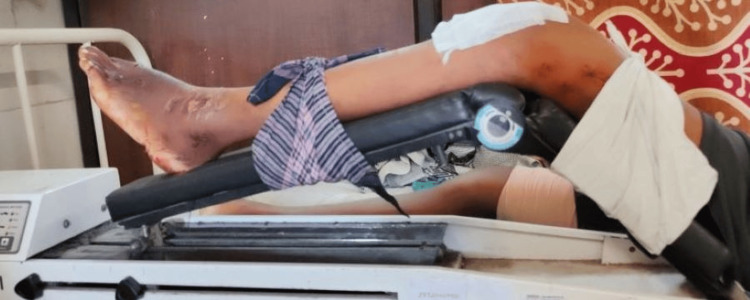
CPM is given to increase ROM CPM: Continuous passive movement; ROM: Range of motion

By this time, the therapist started giving passive ROM or continuous passive movement (CPM) (Figure 3) to the knee joint. Resisted isotonic and isokinetic exercises to the knee and hip were started. Walker was used to accomplish ADLs. With the help of a four-point gait and walker, full weight bearing on the affected side was initiated. Other exercises given were straight leg raise, which helped to strengthen the quadriceps, which in turn helped to stabilize the knee and hip flexors; bridges, to increase the strength of gluteal muscle, which helped to stabilize and support the muscles when the patient walks, climbs stairs and stands up; clamshells, specifically for the gluteus medius muscles that maintain balance while walking and direction changing; step-ups, to target large number of muscle groups that increases overall strength of the leg which helps to lift the leg and bear weight.

Follow-up and outcome measures

Following a four-week course of therapeutic intervention, a subsequent assessment was conducted. Tables 3, 4 show post-rehabilitation ROM and manual muscle testing (MMT), respectively. The outcome measures were taken before and after rehabilitation, which included the lower extremity functional scale and the numerical pain rating scale (NPRS) (Table 5). The scores of outcome measures were found to be increased post-rehabilitation.

**Table 3:** ROM (in degrees) ROM: Range of motion

Joint	Pre-rehabilitation		Post-rehabilitation	
	Left	Right	Left	Right
Knee flexion	0°-15°	0°-120°	0°-60°	0°-120°
Knee extension	0°-10°	0°-120°	0°-60°	0°-120°
Ankle plantar flexion	0°-30°	0°-45°	0°-35°	0°-40°
Ankle dorsiflexion	0°-15°	0°-25°	0°-20°	0°-25°

**Table 4:** MMT MMT: Manual muscle testing; NA: Not Assessable

Muscles	Pre-operative		Post-operative	
	Left	Right	Left	Right
Hip flexors	NA	4/5	4/5	5/5
Hip extensors	NA	4/5	4/5	5/5
Hip abductors	NA	4/5	4/5	5/5
Hip adductors	NA	4/5	4/5	5/5
Knee flexors	NA	4/5	4/5	5/5
Knee extensors	NA	4/5	4/5	5/5
Ankle dorsiflexors	NA	4/5	4/5	5/5
Ankle plantar flexors	NA	4/5	4/5	5/5

**Table 5:** Outcome measures NPRS: Numerical pain rating scale; LEFS: Lower extremity functional scale

Outcome measures	Pre-rehabilitation	Post-rehabilitation
NPRS	9/10	5/10
LEFS	10/80	48/80

## Discussion

Trauma like falls or degenerative diseases can cause distal femur fractures, which frequently need surgery to stabilise the bone [16]. For those suffering from distal femur fractures or other musculoskeletal conditions connected to the distal femur, physiotherapy can be extremely important to their rehabilitation and recovery. According to the study conducted by Kyunghoon et al., essential elements include nutritional support, training in ADLs, community-level rehabilitation, early ambulation, weight-bearing exercises, and the management/prevention of comorbidities [17]. Physiotherapy can be beneficial in a number of ways, including pain management, muscle strengthening, gait training, and functional activities. The study by Janice et al. claims that early mobilisation, gait training, and other treatment modalities are crucial because they support the maintenance or restoration of possible impairments [18]. In order to treat and help patients recover from comminuted distal femur fractures, physiotherapy is essential. A well-designed physical therapy regimen is necessary to assist patients in regaining their function, strength, and mobility. An intervention in physical therapy can help one become more confident. In a study, Paterno et al. reported that intramedullary nailing of femur fractures consistently resulted in healing [19]. However, after surgery, functional limitations and impairments often persist for more than a year, making it challenging for the patient to return to their regular daily routine, gait, or employment. Soft tissue injuries include abrasions, avulsions, and lacerations. Additionally, physiotherapy aids in patients' confidence-building and functional status improvement [20].

## Conclusions

The findings from this case report suggest that physical therapy interventions, including gait training exercises and strengthening exercises, can significantly enhance the mobility, strength, and coordination of patients undergoing rehabilitation for comminuted distal femur fractures. The implementation of these interventions should be overseen by physical therapists and tailored to address the individual needs and goals of each patient. Notably, physiotherapy resulted in improved lower limb ROM, enhanced muscle strength, and increased functional independence in our patient.
